# Patient perspective on pre-operative communication; a post-operative cross-sectional survey of patients with gastro-intestinal malignancy

**DOI:** 10.1016/j.sopen.2026.03.001

**Published:** 2026-03-13

**Authors:** S. Lawday, N. Smart, J.M. Blazeby, C. Metcalfe, A.G.K. McNair

**Affiliations:** a Centre for Surgical Research, Bristol Medical School, University of Bristol, 39 Whatley Road, Bristol, UK; b Department of Gastrointestinal Surgery, North Bristol NHS Trust, Bristol, UK; c NIHR Bristol Biomedical Research Centre, University Hospitals Bristol and Weston NHS Foundation Trust and University of Bristol, UK; d Population Health Science, Bristol Medical School, University of Bristol, 39 Whatley Road, Bristol, UK; e HESRU, Royal Devon & Exeter Hospital, Exeter, UK

**Keywords:** Colorectal cancer, Oesophageal cancer, Regret, Risk communication

## Abstract

**Aim:**

Undergoing major cancer surgery is an important decision for patients. This study assessed the effectiveness of decision-making following colorectal and oesophageal cancer surgery.

**Methods:**

Patients who had undergone colorectal and oesophageal cancer surgery in two UK centres over two years were identified. Validated patient reported outcomes (PROs) were collected by cross sectional survey including perceived confidence in decision-making and effectiveness of risk communication (COMRADE scale), decision regret (Decisional Conflict Score; DCS), and quality of life (EORTC QLQ-PAL15). Uni-variable linear regression was used to explore the relationship between PRO scores and clinical and sociodemographic variables.

**Results:**

Some 143/244 (58%) patients returned the questionnaire. Overall, decision regret was low (median 7.81, range 0–23.4), and perceived confidence in decision-making and effectiveness of risk communication was good (median 95 (75–100) and 85 (70–100) respectively). Higher education level was associated with reduced regret (−0.7; CI −1.2 to −0.1;*p* = 0.016) and a longer length of hospital stay was associated with higher regret (1;CI 0.2–1.9;*p* = 0.014).

**Conclusion:**

Patients perceived decision-making to be effective in this study, but those with adverse outcomes may benefit from further support.

## Background

High quality, patient centred decision-making is a cornerstone of modern healthcare. This involves a partnership between patients and professionals, where treatment options are discussed in the context of patients' preferences using information tailored to the individual [1]. Decision support, ranging from the emotional to practical, should be provided over a multi-stage process with appropriate preference elicitation to enable patients to make adequately informed decisions [2]. Such is the importance of this process, shared decision making now forms part of the NHS constitution and patients' right to make healthcare choices is often protected through legal requirement to gain informed consent [3], [4], [5].

Patient centred decision-making is particularly important before major gastrointestinal (GI) cancer surgery. Surgical resection offers the best chance of long-term survival, but this must be balanced against a significant mortality risk and long-term impact on quality of life (QoL). Colorectal cancer surgery, for example, has an overall survival of approximately 80% after one year, with up to 30% of patient still having significant post-operative symptoms at 5 years [6], [7]. Patients undergoing operations for oesophageal cancer surgery face similar challenges with significant post-operative mortality and a long term impact on patient QoL [8], [9].

There has therefore been much research focused on improving shared decision-making (SDM) [10]. In the United Kingdom (UK), the MAGIC (MAking Good Decisions In Collaboration) programme used quality improvement methodology to demonstrate the feasibility of sustained improvement in SDM in primary and secondary care [11]. It concluded that successful implementation relies on a combination of interventions that support organisations, clinicians, and patients. Cultural change was required so that clinicians view SDM as usual practice and as a fundamental component of safe, effective, and compassionate healthcare. This work, however, did not specifically consider the care of patients undergoing major GI cancer surgery. The aim of this study was to explore perceived communication effectiveness and decision-making regret following surgery for GI malignancy from patients' perspective to provide evidence for the quality of SDM. This will inform the need for further targeted quality improvement work to optimise SDM in GI surgical oncology.

## Methods

This multicentre cross-sectional survey is reported according to STROBE guidelines [12]. Appropriate research ethics regulatory approval was granted (UK NRES Committee London – Bromley 15/LO0617).

### Patients and recruitment

Patients who had undergone an operation with curative intent for management of their colorectal or oesophageal malignancy between October 2014 and October 2016 were included in this study. Patients with metastatic or incurable disease were excluded from this cohort. Purposive sampling of two cancer centres was undertaken to increase diversity of participant characteristics and experience of information provision. University Hospitals Bristol NHS Foundation Trust (UHB) is an urban hospital serving an ethnically diverse population and Gloucester Royal Hospital (GRH) cares for a more rural community. GRH and UHB collectively undertake approximately 120 oesophagectomies and 400 colorectal cancer resections per year. Sequential eligible patients were contacted by post between 01/05/2016 and 31/11/2016. This contained a questionnaire, an information leaflet, consent form and stamped address envelope. Patients who did not reply were re-posted the questionnaire and information at 6 weeks; no further contact was made following the postage of the second questionnaire.

### Outcome measures

Patient reported outcomes measure were collected using validated instruments including COMRADE (combined outcome measure for risk communication and treatment decision making effectiveness) [13], Decisional Conflict Scale (DCS) [14] and the European Organisation of Research and Treatment of Cancer QLQ-PAL15 questionnaire [15].

The COMRADE is a 20-item questionnaire, which measures satisfaction with communication and confidence in decision. Respondents use a 5-point Likert scale from 0 (strongly disagree) to 4 (strongly agree). The DCS is a 16-item questionnaire with five subscales: informed, values clarity, support, uncertainty and effective decision, which uses a 5-point Likert Scale. QoL was measured using the short form of the validated European Organisation of Research and Treatment of Cancer (EORTC) QLQ-PAL15 questionnaire. This 15-item questionnaire assesses health related QoL in terms of physical and emotional function, pain, fatigue, nausea/vomiting, appetite, dyspnoea, constipation, sleeping difficulties and overall QoL on a 4-point Likert scale. This version was selected following feedback because patients may be in an end of life phase and to reduce the burden of the questionnaire.

Clinical and socio-demographic data were collected including age, sex, educational level, marital status, employment status, disease type, treatment type and health related QoL.

### Sample size and analysis

A sample size of 94 patients with oesophageal cancer and 94 patients with colorectal cancer would allow a true difference in mean DCS score between the two groups of 0.4 standard deviations to be detected with 80% power at the 5% significance level.

Data were inputted into an electronic database and analysed using STATA version 15 [16]. Wilcoxen rank-sum was used for binary variables, Kruskal-Wallis for categorical and Spearman's for ordered variables. Multivariable linear regression was not used to tests associations due to abnormal data skew following discussion with medical statisticians.

## Results

One hundred and forty-three patients who had undergone surgical treatment for colorectal or oesophageal cancer questionnaires were returned, with a response rate of 58.6% (143/244 questionnaires returned). Demographics are presented in Table 1. Most patients are male (69.93%) and are aged over 60 years old (85.32%). There was an even split between geographical sites, though there were more patients who had received treatment for colorectal cancer (58.74%).

**Table 1:** Respondent demographics.

	N	%
Gender		
Male	100	69.93
Female	43	30.07

Education		
School/further education^§^	75	52.45
Higher education	33	23.07
Other	24	18.18

Age		
20–39	3	2.10
40–59	18	12.59
60–79	108	75.52
80–99	14	9.79

Marital status		
Single	33	23.07
Relationship	110	76.92

Employment status		
Full time	24	18.18
Retired	110	76.92
Other	5	3.50

Adjuvant or neoadjuvant CRT		
Yes	82	58.16
No	59	41.84

Cancer site		
OG	59	41.26
CR	84	58.74

Place of treatment		
Bristol	71	49.65
Gloucester	72	50.35

Stage		
I	44	32.84
II	48	35.82
III	37	27.61
IV	5	3.93

Median DCS was 7.81 (Q1-Q3 0–23.4) and is presented in Table 2, indicating low decisional conflict. Median COMRADE Confidence with Decision score was 95 (Q1-Q3 75–100) and Risk communication score was 85 (Q1-Q375–100) (Table 2), indicating high confidence in decision making and good risk communication. Median QoL on the EORTC QLQ-PAL15 was 85.7 (Q1-Q3 71.4–85.7) (Table 2). EORTC QLQ0PAL15 subscales are available in Supplementary Table 1.

Most patient clinical or demographic factors did not have an impact on DCS or COMRADE scores after univariate analysis (Table 3). A greater post-operative length of stay is associated with increased DCS. Higher education and marital status were associated with a decreased DCS. An increased length of stay was associated with a decreased COMRADE risk score (Table 3). Patients with stage 3 & 4 cancer had a higher risk communication score and patients without a stoma had a lower risk communication score (Table 3).

**Table 2:** Quality of life, comrade and DCS score.

DCS	Median	Lower quartile	Upper quartile
Total	7.8125	0	23.4375

Subscale			
Uncertainty	0	0	25
Informed	16.6667	0	25
Values clarity	8.333334	0	25
Support	8.33334	0	25
Effective decision	6.25	0	25

COMRADE			
Confidence with decision	95	75	100
Risk communication	85	75	100

**Table 3:** Univariable analysis of explanatory variable for DCS, COMRADE risk and COMRADE confidence.

		DCS	COMRADE	
			Risk communication	Confidence with decision
Kruskal-Wallis		P value	P value	P value
Length of stay		0.0061^a^	0.0072^a^	0.0294^a^
Age groups		0.22	0.1091	0.4321
Wilcoxon		P	P	P
Cancer location	OG^b^			
	CR	0.8196	0.7283	0.9848
Hospital site	Bristol^b^			
	Gloucester	0.4881	0.6128	0.8464
Gender	Male^b^			
	Female	0.4138	0.7635	0.6975
Education	School/further education^b^			
	Higher education	0.0171^a^	0.4079	0.1844
Relationship status	Single^b^			
	Relationship	0.0203^a^	0.0809	0.0204^a^
Employment	Working^b^			
	Not working	0.7814	0.9979	0.8906
CRT	Yes^b^			
	No	0.4867	0.4652	0.3677
Stoma	Yes^b^			
	No	0.1047	0.1386	0.1791
Stage (dichot)	I/II^b^			
	III/IV	0.0036^a^	0.0151^a^	0.089
Spearman		P	P	P
Age		0.1326	0.1421	0.5836
QoL		0.0002^a^	0.0179^a^	0.0014^a^

OG, oesophago-gastric; CR, colorectal; CRT, chemoradiotherapy.

a Statistically significant.

b Control variable.

## Discussion

This multicentre study of patients who had major oesophageal and colorectal cancer surgery found that they had low decisional conflict regarding their choice to undergo surgery, felt high confidence in their decision and that risks had been adequately explained. This suggests that SDM may be perceived to be successful. Longer length of hospital stay was associated with higher decisional conflict, but the effect size was small. Similarly, lower QoL was associated with reduced confidence in decision making. These findings may relate to patients' postoperative experience. For example, patients who experience surgical complications have longer hospital stay and lower QoL. It is plausible that poor surgical outcomes may influence patients' perception of the decision making process but further data is required to explore this further. Higher education was associated with lower decisional conflict, and this may reflect greater empowerment to make meaningful contributions to the decision making process. Increased confidence in risk communication in patients with a stoma and with stage 3 or 4 cancer may reflect the higher risk of surgical intervention and therefore the increased pre-operative discussions that may then have taken place.

A strength of this two-centre study was the use of validated patient-reported outcome measures, but there were some weaknesses. Response rates were approaching 60% which, while similar to other cross-sectional surveys of cancer survivors, indicates the likely presence of responder bias. It is possible that non-responders had worse postoperative experiences, confidence in decision-making and higher conflict; those who died or had recurrence were not included in this study and the results form this cohort are likely to be very different. These results may therefore overestimate patients' satisfaction with decision-making and results must be interpreted in this context. This may also explain the lack of an expected different between patients who had undergone surgery for oesophageal cancer compared to those who had surgery for colorectal cancer, given the difference is clinical and oncological outcomes between these two approached. Generalisability is therefore limited to this patient group and due to the methodology used, the demographics and clinical outcomes of non-responders cannot be ascertained. There is also an unavoidable time lag between patients undergoing surgery and publication of these results, largely related to the COVID pandemic, which must also be considered when looking at the results from this study.

The DCS score has been used in other studies in patients with colorectal cancer; these were used to assess decision aids in elderly patients and the decision about operative approach in rectal cancer, however these were as outcome measures assessing an intervention and not to assess patient opinion of the effectiveness of shared decision making [17], [18], [19]. Wu et al. defined low decisional regret as <25 and our median score was significantly below that at 7.8. The COMRADE score has also not previously been used in this context though has been used within general practice and radiology [20], [21].

SDM comprises different aspects; Bomhof-Roordink et al. highlighted various aspects of SDM in their systematic review; decision making, patient preferences and tailoring information are both vital components of SDM and have been reflected in the measures used in this study [22]. SDM, although heavily supported by evidence and policy, does not always play an integral role within the clinical pathways of hospital and implementation of this has been challenging [11]. This retrospective data has highlighted low regret and high confidence in decision making, prospective live monitoring of SDM would allow clinicians to integrate this into their daily clinical practise. Evidence suggests that integration of SDM into practise may reduce decisional regret and therefore a shift to SDM based decision making model may be required [23], [24]. This could be further targeted at higher risk patients who may be at greater risk of poor clinical outcomes; this strategy is currently undergoing evaluation as part of a wider programme of SDM work [25].

On balance our overall conclusion was that patients have low decisional conflict regarding surgical management for colorectal and oesophageal cancer and were happy with the communication received pre-operatively from clinicians. This reflects largely successful SDM, however must be seen in the context of a low response rate and inevitable survivor bias. Further work regarding the use of core information sets to reduce DCS and COMRADE score could be considered.

## CRediT authorship contribution statement

**S. Lawday:** Writing – review & editing, Writing – original draft, Project administration, Formal analysis, Data curation. **N. Smart:** Writing – review & editing, Supervision, Methodology, Conceptualization. **J.M. Blazeby:** Writing – review & editing, Supervision, Funding acquisition, Conceptualization. **C. Metcalfe:** Writing – review & editing, Supervision, Formal analysis. **A.G.K. McNair:** Writing – review & editing, Supervision, Methodology, Funding acquisition, Formal analysis, Data curation, Conceptualization.

## Synopsis

Patients who have undergone surgery for colorectal cancer or oesophageal cancer in the UK returned questionnaires which demonstrate low levels of decisional regret and high scores for pre-operative risk communication and confidence in decision making.

## Ethical approval statement

Research ethics regulatory approval was granted (UK NRES Committee London – Bromley 15/LO0617).

## Funding sources statement

This research is supported by 10.13039/501100000691Academy of Medical Sciences. AM is funded by a Clinician Scientist Fellowship (NIHR-CS-2017-17-010) from the 10.13039/501100000272National Institute for Health and Care Research (NIHR). SL received a 10.13039/100006662NIHR doctoral research fellowship (NIHR303276). This study was supported by the National Institute for Health and Care Research 10.13039/100015250Bristol Biomedical Research Centre. The views expressed are those of the author(s) and not necessarily those of the NIHR or the Department of Health and Social Care.

## Declaration of competing interest

The authors declare that they have no known competing financial interests or personal relationships that could have appeared to influence the work reported in this paper.

## Appendix A Supplementary data

Supplementary Table 1 Quality of life data. Supplementary Table 1
