# Supplementary material for: Patient perspective on pre-operative communication; a post-operative cross-sectional survey of patients with gastro-intestinal malignancy
Source: Surg Open Sci. 2026 Mar 13;31:33–6. doi: 10.1016/j.sopen.2026.03.001 (PMC13010928; doi:10.1016/j.sopen.2026.03.001)
Supplement: Supplementary Table 1 — Quality of life data. [file mmc1.docx]

Supplementary Table 1 – Quality of Life Data

| Quality of Life | Median | Lower Quartile | Upper Quartile |
| --- | --- | --- | --- |
| Global QoL | 85.71 | 71.43 | 85.71 |
|  |  |  |  |
| Symptoms |  |  |  |
| Physical | 0 | 0 | 8.34 |
| Emotional | 0 | 0 | 25 |
| Fatigue | 25 | 12.5 | 37.5 |
| Nausea and Vomiting | 0 | 0 | 25 |
| Pain | 0 | 0 | 12.5 |
| Dyspnoea | 0 | 0 | 25 |
| Insomnia | 25 | 0 | 25 |
| Appetite | 0 | 0 | 25 |
| Constipation | 0 | 0 | 25 |
